# Integrated community-based dementia care: the Geriant model

**DOI:** 10.5334/ijic.2248

**Published:** 2015-09-23

**Authors:** Ludo Glimmerveen, Henk Nies

**Affiliations:** Department of Organization Sciences, VU University Amsterdam, Amsterdam, The Netherlands; Organization and Policy in Long-term Care, VU University Amsterdam; Executive Board, Vilans, The Netherlands

**Keywords:** dementia care, multidisciplinary teams, organizational form, complex needs, flexibility, levels of integration

## Abstract

This article gives an in-depth description of the service delivery model of Geriant, a Dutch organization providing community-based care services for people suffering from dementia. Core to its model is the provision of clinical case management, embedded in multidisciplinary dementia care teams. As Geriant's client group includes people from the first presumption of dementia until they can no longer live at home, its care model provides valuable lessons about how different mechanisms of integration are flexibly put to use if the complexity of clients” care needs increases. It showcases how the integration of services for a specific sub-population is combined with alignment of these services with generalist network partners. After a detailed description of the programme and its results, this article builds on the work of Walter Leutz for a conceptual discussion of Geriant's approach to care integration.

## Introduction

Geriant is a Dutch organization providing community-based care services for people suffering from dementia. Core to its model is the provision of clinical case management, embedded in multidisciplinary dementia care teams. The organization provides clients and their informal caregivers with care and support from the first presumption of dementia until the client moves to a care home or deceases. Geriant's services are provided in addition to-and in collaboration with-the services of its network partners, including general practitioners, hospitals, care homes, nursing homes, home care and social care organizations, taking a proactive role in aligning services across providers. The organization aims to contribute to a supportive environment that enables people with dementia to live at home for longer, while sustaining the quality of life of both its clients and their informal caregivers. Its model of community-based dementia care has been highlighted as a good example of integrated practice in the Netherlands [1,2].

This article gives an in-depth description of Geriant's approach to care, discussing how their services are organized and what results of their work can be discerned. Emphasis is put on how care integration takes place, both within their organization and across the broader provider network. The descriptions of Geriant's work are based on document analysis (including work manuals, publications by Geriant and third-party publications about Geriant), interviews with key organizational actors (director, physician, case manager), observations of multidisciplinary meetings and the shadowing of a case manager. An initial manuscript of this article has been presented to key stakeholders at the organization for their feedback and input.

In order to extract more general insights from this individual case, this article concludes with a discussion of Geriant's integrated care model on the basis of Walter Leutz's seminal work on his ‘laws of integration’ [3,4]. As Geriant's client group includes people from the first presumption of dementia until they can no longer live at home, its care model provides valuable lessons about how ‘integrative structures’ can be available at different levels of organizing and are flexibly put to use if the complexity of clients’ care needs increases. Its mechanisms of integration develop according to the changing characteristics and needs of clients and their informal caregivers. In addition, the Geriant model showcases how the integration of services for a specific population is combined with alignment of these services with the work of generalist network partners.

The following sections describe the background of the organization's care programme, the services it delivers and the way care integration is organized. After discussing the results that can be discerned from Geriant's work, we use Leutz's work as the starting point for a conceptual discussion of their model.

## Programme background

Geriant provides its services in the northern part of Noord-Holland province, a semi-urban area around 30 km north of Amsterdam covering around 1000 km^2^. The population in this area counts slightly over 600,000, with around 7% over the age of 75 and around 2% over 85 [5]. An estimated 8029 people in this area suffered from dementia in 2011, which is expected to increase with 164% by 2040. Within this area, Geriant's community-based programme provides care services to all people with dementia who still live at home, ideally from the first signs of cognitive decline until they move to a nursing home or decease at home.

In 2000 Geriant started as a joint programme of the nursing homes and public mental health care organization in the region, becoming an independent legal entity in 2004. The programme was established because services for people living at home suffering from dementia were believed to be fragmented and of insufficient quality. Care was provided along organizational lines and public funding schemes, not matching the complex, ‘messy’ and often unpredictable situation of the target group. Medical diagnosis and treatment were separately organized from the provision of care [6]. Services were confined to diagnostics and crisis intervention and too often resulted in premature nursing home or hospital admission. While facing a sharp growth in the prevalence of dementia in the community, Geriant was established to improve the capacity, quality and alignment of acute and long-term care for people living at home with dementia.

Geriant's integrated model of community dementia care was jointly developed by these founding organizations. In the early phases of the initiative, these organizations made their staff available and channelled public funding to the programme. The regional care purchasing office (the institute contracting providers for the mandatory universal insurance for long-term care) provided both regulatory and financial support in this process. When the organization became an independent legal entity in 2004 its governance structures were still interwoven with its founding organizations, who were members of its supervisory board. Although such close connections provided a crucial support base that enabled the organization to operate effectively, it also made its operational and governing structures rather complex. For example, Geriant had to make arrangements with the HR-departments of all organizations that assigned staff to the programme, while its autonomy in recruitment was also compromised. It had to deal with nursing homes for daily operations (e.g. for client placement), while at the same time these were part of its supervisory board. In the years after becoming an independent organization, the founding organizations’ influence in Geriant's administrative structures was reduced and eventually ended. Close operational ties remained.

While initially funding was channelled through its founding organizations and came from various public funding schemes, Geriant's activities are now funded through the mental health component of the national health care insurance. This insurance is mandatory for everyone living in the Netherlands, with the content of its basic package defined by the national government. Shrinking public budgets for mental health care resulted in a 7% decrease in Geriant's 2012 budget compared to the year before.

Geriant has contractual agreements with health care insurers that are negotiated on annual basis. These contracts include details on bundled payments and service specifications for the forthcoming year, with the nature and scope of activities being specified in so-called ‘diagnostics-treatment combinations’. The contracts also specify quality standards and procedures, whereby Geriant needs to report to insurers on monthly basis.

At the end of 2012 Geriant served 3536 clients in its community-based programme, having 174 people (124 full-time equivalents) on its payroll. The annual enrolment rate has steadily increased over the years as a result of more referrals by general practitioners, with an average annual growth rate of almost 12%. On average the period between enrolment and moving to a nursing home or deceasing is around 2 years, although with large variance.

Since 2011 Geriant also implements a case management programme in all nursing homes in the region. In this programme a case manager is based in a nursing home, providing services to its residents (who all have their own general practitioner), while supporting and training the nursing home staff in working with people suffering from dementia. This case study, however, focuses on those services provided for clients living at home. Their model for community-based care has been implemented for over 14 years now and is well-documented.

## Model of service delivery

Geriant provides an integrated set of dementia care services that includes diagnostics, clinical case management and treatment. At the core of the organization's services are the so-called *DOC-teams*. DOC is a Dutch abbreviation that stands for ‘Dementia Assessment and Case management’. These multidisciplinary teams provide a set of dementia-related acute and long-term care services throughout clients’ care trajectory. In all its four sites Geriant houses a multidisciplinary DOC-team, consisting of case managers (around 12 per team), a social geriatrician (at least two per team), a psychiatrist, clinical psychologist, dementia counsellor and one or more specialized home care nurses. Throughout the care trajectory, a case manager is the client and informal caregiver's focal point and supports them in coordinating care services, while also providing treatment or counselling itself. For specialized or intensive treatment, the case manager can call upon the other specialists in the team. All members of the multidisciplinary teams are all employees of Geriant.

In case more intensive treatment or observation is required, Geriant's DOC-centre serves as a closed short-stay clinic with 16 beds where clients usually stay for a few weeks. During the last phase of their stay it is assessed whether a client can return to his or her own home or whether admission to a nursing home needs to be arranged. The short-stay clinic houses a multidisciplinary team with capacity for 24-hour clinical care.

**Table:**
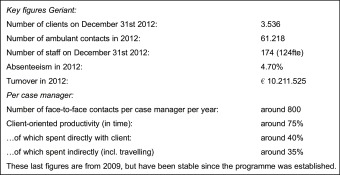

### The care trajectory

All clients are referred to Geriant by their general practitioner, the gatekeeper in the Dutch health care system, at the onset of dementia. In case dementia is diagnosed people can be enrolled in the programme and Geriant is eligible for funding through clients’ health insurance. Without a diagnosis of dementia people cannot be enrolled at Geriant's programme.

Generally around two or three weeks after a general practitioners’ referral, intake and diagnostics are performed jointly by a case manager and the team's social geriatrician. An assessment of the client's physical status is combined with a first identification of care needs. This can take place at people's homes or in the DOC-centre. During the intake the case manager interviews both the client and his or her informal caregiver on the development of their situation. Questionnaires are used to test the client's cognitive capacities. The initial care needs are discussed straight away. The physician performs a physical assessment and laboratory tests. Based on the information that is gathered, the diagnosis is discussed and formalized in the weekly meeting of the multidisciplinary team, whereby the social geriatrician has the final say. Diagnosis is based on nationally acknowledged professional guidelines for diagnosis and treatment of dementia. In the very likely case of a positive diagnosis (over 95% of all referrals), the case manager meets the client and his or her family (or other informal caregivers) to discusses the diagnosis. Normally the team's social geriatrician or psychologist joins this meeting.

After the diagnosis is shared with the client and informal caregiver(s), a more detailed analysis of care needs is carried out by the case manager, client and informal caregiver. This analysis is done using a model for the structured mapping of clients’ situation. This model has been developed by Geriant and its founding organizations, as the instruments available at that time were regarded insufficient for ambulant dementia care. It consists of eleven ‘care dimensions’ that reach beyond the clinical level, including issues related to the available informal care (two dimensions) and the client's broader network and home environment (three dimensions). Based on this assessment, the case manager, client and informal caregiver jointly identify the two or three most important challenges, formulate realistic objectives and integrate these in the client's care plan. The outcomes of this meeting and the individual care plan are again discussed in the DOC-team's weekly meeting, after which service delivery and case management can start.

During the care trajectory the client's condition and care package are evaluated at least on annual basis. Assessments of clients’ cognitive capacities are repeated and the client and informal caregiver are asked about their satisfaction with the care provided. The case manager, client and informal caregiver jointly renew the plan for care and treatment for the next year, which is discussed by the case manager with the team's social geriatrician. The general practitioner, who remains responsible for clients’ primary care needs, is notified in writing.

When a client's condition escalates into an acute behavioural crisis (e.g. extreme paranoid or suicidal behaviour), care crisis (e.g. when an informal caregiver suddenly becomes unavailable) or medical crisis (e.g. a delirium), caregivers can contact the case manager during office hours, while outside office hours Geriant has an agreement with the emergency psychiatric department of the regional mental health organization. In crisis situations the case manager visits a client at home, preferably together with the physician and in the presence of informal caregivers and/or family members. They assess acute problems in the client's physical condition and care delivery, whereby immediate short-term support and treatment are discussed and decided upon. Before and after the home visit, the case manager contacts the client's general practitioner (and relevant other network partners) to gather and share information.

While most of the crisis situations can be managed at home, sometimes admission in a nursing home, the short-stay clinic or a hospital is required. In each of Geriant's four sites a case manager doubles as a placement coordinator for the local nursing homes. Their ability to perform triage of the team's clients is recognized by these nursing homes, allowing them to put urgent cases on top of the waiting list or make use of the spare emergency bed that the nursing homes alternately have available. In case of acute behavioural or (relatively non-complex) physical problems, admission in the short-stay clinic can be decided upon by the DOC-team's and clinic's social geriatricians. In case of a more complex condition, the team's social geriatrician arranges hospital admission.

Throughout the care trajectory, client's informal caregivers are explicitly included as a crucial target group of Geriant's activities. They are not just seen as a resource, but approached as co-clients who might also need counselling, training and support. Geriant acknowledges that overburdening of informal caregivers, even in the initial stages of the clients’ care, is one of the main reasons for admission to a nursing home [7,8]. In addition, informal caregivers are engaged as partners when planning care in order to tailor services to clients’ specific context. For example, whenever possible family members or other informal caregivers are included in the inter-organizational care teams that are formed around individual clients’ with a complex and unstable condition (discussed later). In their various roles, informal caregivers are included in the care trajectory from the beginning.

A comprehensive client flow chart is included in Annex I.

### Approach to case management

While there is an ongoing policy debate on whether case management should be initiated as early as possible or only when the client's condition starts deteriorating [9], Geriant deliberately chooses the first option. According to Geriant's vision, case management starts preferably at the first signs of dementia in order to prevent the ad hoc crisis interventions that used to characterize community-based dementia care in the region. In practice this sometimes means that in the initial stage, when most clients and caregivers still function relatively independently, case management entails no more than an annual phone call.

Geriant's approach to case management is characterized by its integration in the multidisciplinary DOC-teams, as well as the clinical nature of case managers’ tasks: they do not only coordinate, but also perform care tasks themselves. This is referred to as clinical case management, being different from a more narrow ‘brokering’ model in which they only align and coordinate care services provided by others. At Geriant case managers integrate the role of coach, care broker and medical practitioner.

Case managers maintain regular contact to monitor the situation of their client and informal caregiver. Depending on the client's situation, this can range from a weekly home visit to an annual phone call. They counsel their clients and informal caregivers to address problems themselves if possible and give them a sense of support even when there is no acute need. If required they arrange services at other network partners and stay in contact with them, aligning care planning and sharing clients’ feedback on the quality of services. If relevant, home care and social care providers are instructed to signal and communicate changes in clients’ situation, or to provide practical support with e.g. taking medication.

In addition to these coaching and coordination tasks, case managers can provide dementia-related treatment and care themselves and call upon the specialists in their team when their involvement and expertise is required. Depending on the situation, members of the DOC-teams can provide behavioural, pharmaceutical or system-oriented (usually the client and his or her family) treatment. This mainly includes cognitive behavioural therapy and motivational interviewing (either by the case manager individually or together with the psychologist), psycho pharmaceutical treatment (by the team's physician) and training for informal caregivers or practitioners in the network about dealing with symptoms of dementia. The training programmes for informal caregivers are also offered online.

The current average case load per full-time case manager is 70 clients plus their informal caregivers. Although this is above the consensus-based national guideline of 50 and indeed experienced as a high burden by the case managers, it should be noted that the work load per client varies extensively. This mixed character of the case load should be taken into account when interpreting this figure.

### Care integration

To ensure care continuity and alignment across services, a range of integrative mechanisms can be identified within and across Geriant's organizational boundaries, both at strategic levels and in professionals’ daily care practices. Whether such mechanisms are indeed put to use depends on what the client's condition requires; when case management entails one phone call every few months, requirements for alignment are less pressing compared to highly unstable and complex situations.

Looking at coordination of care within the organization, the multidisciplinary character of the DOC-teams entails that most care coordination takes place within the daily operations of these individual teams, with relatively little ‘inter-departmental’ coordination required. All case managers have thorough background knowledge on dementia and can perform basic treatment and care themselves, communication about clients’ medical status is made much easier and the specialists of the team do not have to be called upon at every instance. Throughout the teams’ work, the case managers are the prime source of information on clients’ situation, while the team's social geriatrician has final responsibility for care that is provided.

In all four sites, the team members attend a weekly multidisciplinary meeting to discuss diagnostics, care analysis, planning, evaluations, complications and care transitions of clients. As case managers spend most of their time ‘outside’ in the community, these meetings are the main opportunity for alignment in the teams. Besides these meetings, case managers present and discuss individual clients with the social geriatrician after annual evaluations or more often if the client's situation requires so.

All clients’ records are kept in electronic health files that were introduced in 2011 and fully replaced paper files. These are available for all practitioners from the various disciplines in the organization. Currently it is not yet possible for external practitioners to gain access to these files. In future Geriant hopes to acquire a portal structure through which relevant network partners as well as clients and their informal caregivers can access these files. How all this should be organized, both content-wise and with regard to the ICT-infrastructure that is required, is still a challenge.

Case managers also play a central role in coordinating activities with external network partners. The relationship with general practitioners is crucial here. In their role as gatekeepers they refer clients to Geriant while remaining responsible for their primary care needs. Geriant is in touch with all general practitioners in its catchment area (around 340 in total). Usually they are updated in writing after diagnostics, care transitions and evaluations, while during office hours general practitioners can call straight to the DOC-teams’ physicians for consultation. Geriant has recently aligned case managers’ caseload according to clients’ general practitioner, enabling them to periodically meet and discuss all their clients at once.

Furthermore, case managers support clients and informal caregivers in applying for other (home) care services. They instruct home care and social care workers to identify and communicate changes in clients’ situation, keep in touch with them on regular basis and make joint visits if possible. If possible, evaluative meetings with other providers are arranged around twice a year, preferably in the presence of the client and informal caregiver. If complaints about care services cannot be satisfactorily addressed, case managers support clients to arrange services with another organization.

In case a client's condition turns highly unstable and complex, inter-organizational teams can be established around individual clients. The formation of such ‘Dementia HomeTeams’ (‘ThuisTeam Dementie’) is an initiative of the regional dementia platform, in which Geriant participates together with local housing, social care, home care and patient interest organizations, as well as the regional care purchasing office and municipalities. When such an inter-organization team is established, preferably all people directly involved with the client (including informal caregivers) meet every two or three months, taking a joint care plan as a starting point. In these periodic meetings, all parties involved share experiences, discuss and evaluate the client's situation and care services provided, while jointly exploring possible solutions to emerging challenges. Generally, these cross-network multidisciplinary teams are only formed for less than 10% of all clients; for more stable, ‘straightforward’ clients less intensive care integration is required and thus applied.

Although now challenged by changing legislation (which is discussed later), Geriant does not explicitly categorize its clients according to the complexity of their situation, meaning that caseloads have a very heterogeneous character. Case managers often manage without much interference from their team's specialists; a social geriatrician stated that she was only actively involved with around a quarter of her team's clients after diagnostics have taken place. While there is an infrastructure for care integration in place for all clients, the extent to which these structures are indeed put to use depends on what a client's situation requires.

In case people can not stay at home any longer, Geriant's case managers have a central role in their potential nursing home admission. As their ability to perform triage is recognized by these nursing homes, one case manager in each team is granted the role of nursing home placement coordinator.

### Dealing with system-level barriers

While currently 95% of Geriant's activities are funded through the same funding scheme (the national semi-public mandatory health care insurance), the organization still experiences challenges to integral service delivery within this scheme. While in some instances the organization tries to work around such system-level boundaries, it is also involved in lobbying campaigns in attempts to structurally overcome them.

Initially, only services provided directly to clients were covered by the insurance scheme. For this reason informal caregivers were also enrolled as clients, making them eligible for support. This improvised solution is not necessary anymore in the current funding system; now one budget is available after diagnosis that covers all Geriant's services.

A number of challenges have recently emerged as a result of developments within the funding system. First of all, until recently the organization only had to negotiate annual contracts with one insurance company who acted as a representative for all others. In order to stimulate competition among these insurers, this representational model has been abandoned, meaning that Geriant has to negotiate separate contracts with all insurance companies. This does not only result in different contracts and conditions per insurer, but it also presents them with their diverging views of how dementia care should be organized.

Another challenge to integrated service delivery stems from new regulation for mental health care, which is how Geriant's services are formally classified. Current policies explicitly distinguish basic from specialist mental health care based on clients’ condition and care needs, forcing providers to administer these groups separately and only deliver certain types and volumes of care per category. This introduces a bureaucratic barrier to seamless service transitions, e.g. when people's condition unexpectedly deteriorates or when an informal caregiver suddenly becomes unavailable.

Although Geriant's services are mainly funded from one source, incentive mechanisms across the broader network of providers are fragmented. Incentives for substituting nursing home's services with community programmes are insufficient, as the two are funded through different schemes. The same goes for medical home care and social care services like day care. Geriant has been part of a lobby campaign towards the Dutch government to plead for more integrated funding across the sector, using a study by the regional care purchasing office (discussed later) to support the claim that cost-saving substitution is possible. Currently some funding mechanisms are indeed changing, as from 2015 Geriant's services will be funded through the same scheme as other home care services.

While system barriers such as fragmented funding across providers exist, good working relations with network partners have considerably contributed to alignment of dementia care services. As mentioned before, these relations can be traced back to Geriant's collaborative founding history. Moreover, high-quality service to network partners is seen as key to its success. Strong involvement of a range of network partners in the programme's start-up years, including the regional care purchasing office, has resulted in close informal ties and open communication, also after Geriant turned into an independent organization.

While this collaboration has a largely informal character, Geriant has an ambivalent attitude towards formalizing it. On the one hand the Dutch health care inspection has recently started monitoring care networks in addition to its inspection of individual organizations. From this perspective there is an increasing demand to make agreements explicit and formalize them. On the other hand there is reluctance to actually do so. First of all, the current informal way of collaborating frees all parties from contractual or bureaucratic constraints, allowing flexibility if needed for individual cases. In addition, when formalizing the informal position that Geriant currently holds with regard to, e.g., triage for nursing home admissions, other network actors might feel threatened in their autonomy, reducing their acceptance of each other's involvement in their work.

## Organizational form and governance

Geriant is a horizontally structured organization with its management positioned close to its professionals. The organization has one director to whom the managers of six professional units (four multidisciplinary DOC-teams, the short-stay clinic and the nursing homes team) directly report, in addition to the head of the administrative unit and the chief physician. The director himself reports to the supervisory board. All professional units are led by one team manager. This means that out of 174 employees (end of 2012) 9 people are in formal management positions, 6 of whom are directly in charge of professional units. Clients and employees are represented in a clients’ council and an employees’ council. Members of the client council include informal caregivers of clients as well as representatives of the local departments of Alzheimer Nederland, the Dutch patient interest organization for people with dementia. An organization chart is provided in Annex II.

Geriant explicitly chooses to keep its organizational units small to be able to respond to the unpredictable nature of its clients’ situation. When teams grow in size, they should be split rather than supplemented with an extra management position. The organization's 2012–2015 multi-year plan explicitly states that growth is in itself not a goal. The absence of multi-layered hierarchical structures, as well as the presence of all disciplines in every team, provides these teams with the flexibility and short communication lines that are required for responding to the sometimes unpredictable nature of clients’ situation. All members of the DOC-teams are on the payroll of Geriant, use the same electronic patient file and meet in a weekly multidisciplinary meeting with the entire team that normally consists of not much more than 20 members. With case managers acting as expert linking pins between clients and the DOC-teams, internal coordination of care is performed relatively efficient and effective.

**Table:**
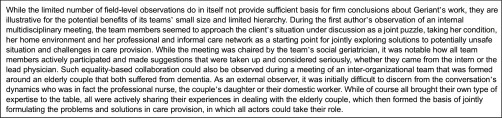

The clinical nature of Geriant's case management has its consequences for the professional qualifications that are required for it. While initially some of the case managers (delegated by the founding organizations) had a background in social work, it was found that sufficient knowledge on dementia and nursing skills were required to perform the broad range of tasks inherent to the position. Geriant now only employs professional nurses as case managers. Every case manager first has to go through a dual one-year training programme in clinical case management for dementia. This programme was developed by Geriant, but has been taken over by a training institute at an academic hospital to be offered to a wider audience.

For clients it is important that case managers are easily accessible and available throughout the week. From a national evaluation [10] it came forward that accessibility can be a problem if case management is not a stand-alone position but part of a wider set of responsibilities. Geriant acknowledges the importance of accessibility, hence case managers are required to work at least 3 days a week and preferably more. Here it should be noted that a relatively high percentage of people in the Netherlands works part-time.

### Quality management

As the available instruments at the time of Geriant's start-up were regarded inappropriate for ambulant dementia care, the organization developed elaborate manuals for case management, medical diagnostics and care needs analysis. These manuals are structured along the eleven care dimensions that were discussed earlier and are shared publicly on the organization's website. Geriant has developed its own quality system that was certified by HKZ, the main certifying body for safety and quality in Dutch health care. This is a requirement stated in the contractual agreements with insurers.

Both the organization as a whole and its individual organizational units have their annual planning cycle. The director meets with the unit managers every month to discuss progress, while a formal evaluation of each unit takes place on annual basis. Throughout the year, 20 indicators provide management information and serve as accountability mechanisms within the organization. This includes the ‘Incident Reporting on Client Care and Organization’ monitor, the continuous client satisfaction survey, the six monthly employee satisfaction survey, the internal and external quality audit and production, and HR and financial indicators.

As Geriant is formally classified as a mental health care organization, it is required to publish annual data on a number of obligatory performance-indicators that are defined on a national level. Since 2012 it implements a Routine Outcome Measuring system that is built on two key instruments. Clients’ condition is assessed using Reisberg's Global Deterioration Scale (or GDS-r), while caregivers’ condition is measured using the EDIZ-scale (a Dutch abbreviation of ‘Experienced Burden from Informal Care’).

## Programme results

Since 2012 Geriant implements its Routine Outcome Measurement system and started measuring client and caregiver's condition using the GDS-r and EDIZ scales. With one measurement at enrolment, at least one intermediate score and one measurement when leaving the programme, these scores provide some insight in the development of a client's condition and its caregiver's well-being along their care trajectory. On a 0–7 scale the GDS-r showed respective measurements of 3.4, 3.8 and 5.0, in line with the progressive nature of dementia and indicating the severe level of cognitive decline when the support of Geriant ends. The 0–9 EDIZ-scale shows respective scores of 4.4, 4.1 and 4.9, suggesting that caregivers feel to some extent relieved of their burden after entering the programme, despite clients’ simultaneously measured deterioration. They experience higher burdens at the end of the programme, mainly when the client is admitted to a nursing home, in line with the notion of informal caregivers’ burden as an important predictor institutionalization. Although more specific data are required to capture the full dynamics of the trajectory, the initial decrease in caregiver burden suggests a positive contribution to a supportive home environment, enabling people with dementia to remain at home for longer and postpone admission to a nursing home.

An important reason for establishing Geriant was to take community-based dementia care beyond its reliance on ad hoc crisis interventions, which often resulted in hospital or nursing home admission. Looking at the 2012 figures, 981 crisis interventions took place. Although more reliable figures are not yet available, an estimated 100–150 of these interventions ended in emergency admission in the DOC-centre or nursing homes. While this shows that crisis situations are mainly dealt with at home, it should be mentioned that data from the situation before 2000 are not available.

For assessing client satisfaction Geriant performs continuous measurements. After a client deceases or moves to a nursing home, informal caregivers are invited to take part in a satisfaction survey that is developed and conducted by an external research institute. In 2012 informal caregivers graded the DOC-team's services with a 8.2 out of 10, similar to results in earlier years. About 95% of the respondents would recommend the DOC-team's services to others (Net Promoter Score).

In a broader national evaluation of case management for people with dementia (Geriant was one out of thirteen initiatives studied), a survey among informal caregivers showed that case management made them better informed and able to deal with symptoms of dementia, more aware of the availability of care and support services and feel less lonely. The same survey showed that case management reduces the number of unplanned visits to client's general practitioner [10].

Assessments of Geriant's network partners’ satisfaction also show positive results. General practitioners graded Geriant's work with a 7.8 out of 10 on average and especially seem to appreciate the combination of diagnostics and case management. The national evaluation discussed above showed that general practitioners that spend relatively much time on dementia care feel relieved after the introduction of case management [10]. The steady annual increase in referrals to Geriant seems to underline this positive assessment. Other network partners graded Geriant's work with a 7.9 out of 10.

In a national ranking of employee satisfaction in 2013 (not confined to care organizations), Geriant came out fourth in the category for organizations below 1000 employees. On average, Geriant employees graded their employer with an 8.3 out of 10 when asked for their general satisfaction, compared to a national average of 7.5 [11].

### Cost efficiency

While Geriant's services cost 8 euro per day per client on average, this figure concerns both clients who receive one or two phone calls per year, as well as those with a complex condition receiving extensive support. It also does not include costs of, e.g., social care or home care services provided by network partners. Yet to find out whether substitution was taking place as a result of Geriant's work, the regional care purchasing office compared the nature and costs of dementia-related care in seven regions in the Netherlands [12]. Geriant's catchment area, the north of Noord-Holland province, was taken as one of these regions.

Looking at the cost of care for those people who were diagnosed with dementia and deceased in 2011, multiple funding schemes for acute and long-term care were reviewed to see whether substitution took place as a result of Geriant's programme. Compared to the national average, three to four times as many people in the organization's catchment area used mental health care services, which is how Geriant's services are formally classified. In absolute figures the dementia-related mental health care costs in this region were more than double the national average, although per user they were by far the lowest (more than a third below the average). So while more people with dementia used mental health care service, figures from other funding schemes suggest how substitution did take place. A group receiving general long-term care but no mental health care services was compared to a group receiving both general long-term care and mental health care services. It should be noted that the latter group did not only consist of Geriant's clients, while also alternative explanatory factors were not fully excluded. This being said, the comparison shows that the total care expenditure in the last life phase was 47% lower for the group also using mental health care services, saving on average more than 48.000 euro per person. In addition, the average length of stay in a nursing home was around 9 months lower for this group, while expenses for dementia-related hospital care were 40% below the national average for people in Geriant's catchment area.[Fig fg0003]

The model developed by Geriant is often referred to as an exemplary practice for community-based dementia care in the Netherlands. In 2005 the Dutch Secretary for long-term care mentioned Geriant in a letter to the Parliament about the National Dementia Programme [2] as a good example of successfully improving coherence and collaboration in care for people with dementia. In 2012 the aforementioned study by the regional care purchasing office raised considerable media attention when suggesting that national implementation of the model would save an annual 200 million euro on public long-term care [12].

## Discussion

Geriant's approach to care shows us how ‘integration’ can have a variety of meanings and entail a wide range of organizational practices. It ranges from loosely linked network partners to closely knit multidisciplinary teams, while it can address an entire care system or the network of an individual client. To distill lessons from the Geriant model and position it within this variety of forms and levels of integration, we take the work of Leutz [3,4] as a starting point for a more conceptual discussion.

A key issue in Walter Leutz's work revolves around the question which extent of integration should be pursued for different target groups, as ‘you can't integrate all of the services for all of the people’ [3, p. 83]. He discusses how more extensive integration is appropriate for people with complex, unpredictable conditions, while ‘simpler approaches’ [4] might suffice for populations with less challenging care needs. Leutz distinguishes three levels of integration with increasing levels of intensity: (1) ‘linkage’, whereby professionals know when and where they can refer people if their condition requires so, (2) ‘coordination’, whereby ‘explicit structures and individuals are installed’ [3, p. 85] to address discontinuities within and between systems, and (3) ‘full integration’, whereby new programmes are established in which resources are pooled and directly controlled for the delivery of integrated service.

Using Leutz's classification, Geriant can be characterized to apply linkage, coordination as well as full integration in organizing its dementia-specific services while aligning these with other, generalist services. In the early stage of their care trajectory, clients are always referred to Geriant by their general practitioners. Such linkage is, e.g., supported by the Geriant website which informs care professionals when, where and how to refer clients at the presumption of dementia. In addition, linkage takes place when case managers refer clients to other providers in medical and social care or instruct clients and their informal caregivers to apply for them. It is only when the need emerges to further align services among these network partners, that more explicit coordination structures are applied. In such cases, developments in clients’ situation are for example discussed with their general practitioners, home care workers are instructed to identify and communicate changes, joint home visits are made, or hospital admission and discharge are coordinated with the hospitals’ physicians. Coordination with network partners can be ‘scaled up’ into further integration if the complexity of a client's condition requires to do so. The formation of inter-organizational teams around these individual clients allows providers, together with clients’ informal caregivers, to work from a shared plan and to jointly address care challenges for these specific, complex cases. Within Geriant itself, more extensive integration takes place in the multidisciplinary DOC-teams, where the work of various specialists is fully integrated into a joint daily practice. In severe cases, this takes place in the context of its short-stay clinic.

That being said, the question still remains which clients qualify for which level of integration. According to Leutz, ‘defining the persons who belong in such [fully integrated] programmes, assembling the necessary services and allocating appropriate resources (enough to do the job but not so much to short-change other groups) are among the most pressing issues in integration policy and practice’ [3, p. 88]. As a guiding principle, Leutz links the required level of integration to the level of complexity of clients’ care needs. At Geriant, clients with very diverse care needs are deliberately brought together in one case mix, with conditions ranging from relatively simple and stable to complex and unpredictable and with integration mechanisms varying from linkage to full integration. It can be hard to predict how clients’ situations evolve, as complexity can emerge unexpectedly from stable situations, e.g. when an informal caregiver gets overburdened and becomes unavailable, or when a client's physical condition suddenly deteriorates. To accommodate such uncertainty, Geriant's care model tailors the level of service integration to the situation at hand within the same organizational setting. As the same clients often make use of dementia care services long enough to require different levels of integration, it is the programme and its organization that follow these clients’ requirements, instead of clients being shifted from one programme to another.

Indeed, Leutz states how his three ‘prototypical’ levels of integration in practice tend to mix, as service providers ‘integrate different aspects of their programmes to different degrees’ [4, p. 7]. Geriant showcases how such a mix can be flexibly applied within the same group of clients. Sometimes case managers go about their work without much involvement of their specialist team members, while in other instances care problems are jointly addressed in their fully integrated multidisciplinary team. Even when clients and their informal caregivers are sufficiently served with a phone call every few months, the ‘templates’ and supporting infrastructure for integration are in place, allowing smooth transitions and cross-boundary practices when more integrated service delivery is required.

While Geriant's goal is to integrate community-based care services for people with dementia, this group constitutes only a sub-population for many of Geriant's network partners. There is a risk that, building on Leutz's words, dementia-specific integration results in generalists’ fragmentation. General practitioners, for example, deal with the care needs of a wide range of patient groups, hence ‘asking them to add special information or actions for small subgroups of patients simply increases the already considerable pressure of their practice routines’ [3, p. 92]. Within their diverse client population, they are confronted with attempts at service integration within various domains and for different sub-populations, e.g. youth or people with diabetes or cardiovascular diseases. Looking at such a wide diversity of integrative efforts, a key question is how specialist care services can be integrated while still accommodating the role of primary care as a linking pin between various specialist domains.

In order to align the work processes and-thereby-the time and effort required by general practitioners for dementia-specific alignment, Geriant decided to restructure case managers’ caseload based on client's general practitioner, enabling them to periodically meet and discuss all their clients at once. Again drawing on Leutz, such sensitivity and support to general practitioners’ practice is important if care integration is indeed to solve operational problems, instead of creating new ones by introducing new kinds of fragmentation [4]. An important foundation for Geriant's success is found in how they bring relief to general practitioners’ burden of coordinating care for people with dementia. Especially if primary care's role as a linking pin between various specialist domains is further emphasized, internal integration of specialist domains should go hand in hand with sufficient alignment with primary care professionals.

The Geriant case shows us how care integration is not about ‘eradicating boundaries altogether’ [4] or finding structural, formal solutions for everything. The programme was established 14 years ago as a result of local leadership, based on the shared commitment of a group of providers to address insufficiencies in the regional provision of dementia care services. While this resulted in newly established organizational entities and formal collaborative platforms with policies that facilitate joint care practices, the organization largely relies on the shared frame of reference that has been developed among the various providers involved, and the informal coordination mechanisms that are built on this [13]. Regularly, such informal coordination seems to be preferred to formalized collaboration, as can be seen in the hesitance to formalize relations between network partners when the health care inspectorate started assessing entire care networks, or when the formal influence of Geriant's founding organizations was disentangled from its governance structures. Trust and good working relations at the local level seem to have played an important role in overcoming system-level fragmentation and dealing with shifting regulatory boundaries that challenge established work processes. The involvement of the regional care purchasing office from the programme's onset has for example been very supportive in dealing with regulatory challenges. The importance of informal coordination mechanisms is also found closer to the primary care process. While the integration of functional structures and processes plays an indispensable facilitating role in service delivery, Geriant's small teams and limited hierarchy enable the informal ‘multidisciplinary matching’ to take place among the various providers involved, taking the client's situation as a starting point to align boundaries if necessary.

## Figures and Tables

**Annex I.**
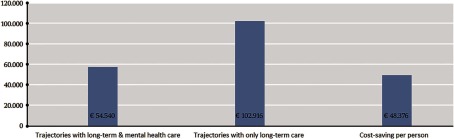
Client flow chart

**Annex II.**
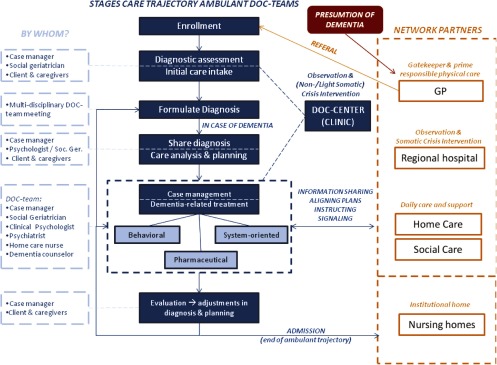
Organizational chart

**Figure fg0003:**
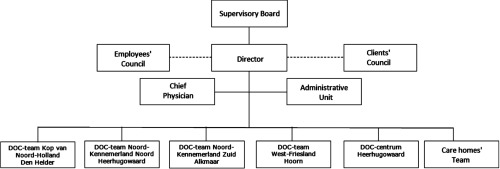
Average care expenses per person in 2008–2010 for people deceased in 2011 with a psycho geriatric condition [12].
